# Risk profiles for mental disorders

**DOI:** 10.1192/j.eurpsy.2021.220

**Published:** 2021-08-13

**Authors:** S. Gülöksüz

**Affiliations:** Department Of Psychiatry And Neuropsychopharmacology, Maastricht University Medical Center, Maastricht, Netherlands

## Abstract

**Abstract Body:**

Prognostication is at the bedrock of clinical practice. In essence, diagnosis aims to inform clinicians for decision-making processes by providing a picture of future events such as course, outcome, and treatment response. To make a better clinical prediction on a case-by-case basis, diagnosis is enriched by individual characteristics and (bio)markers, with the aims of stratifying patients first and ultimately reaching the mountaintop: personalized medicine. However, there are two major obstacles on the road to personalized psychiatry. First, the current psychiatric diagnostic classification system is inadequate for tailoring individualized management plan, let alone for guiding the clinician for diagnosis-specific treatment selection—such that response to the same treatment plan largely varies among patients with the same psychiatric diagnosis, whereas patients with different psychiatric diagnoses benefit similarly from the same treatment protocol. Second, except for a few tests for ruling out other medical conditions, there exists no diagnostic, prognostic, or predictive (bio)marker in psychiatry. Risk profiling is even a more challenging and ambitious goal as early psychopathology is multidimensional, fluid, and pluripotent with heterotypic outcomes that cut across traditional diagnostic boundaries. By acknowledging this complexity and the shortcomings of current taxonomy, the field has recently shifted from risk profiling frameworks that rely on discrete diagnostic categories in isolation for prognostication (i.e. clinical high-risk for psychosis) to transdiagnostic clinical staging models. In this session, I will attempt to discuss where we are at with risk profiling in psychiatry and what steps need to be taken to achieve this ambitious goal.

**Disclosure:**

No significant relationships.

